# Vitiligo: An Autoimmune Skin Disease and its Immunomodulatory Therapeutic Intervention

**DOI:** 10.3389/fcell.2021.797026

**Published:** 2021-12-14

**Authors:** Wei-Ling Chang, Woan-Ruoh Lee, Yung-Che Kuo, Yen-Hua Huang

**Affiliations:** ^1^ TMU Research Center of Cell Therapy and Regeneration Medicine, Taipei Medical University, Taipei, Taiwan; ^2^ International Ph.D. Program for Cell Therapy and Regeneration Medicine, College of Medicine, Taipei Medical University, Taipei, Taiwan; ^3^ Graduate Institute of Medical Sciences, Taipei Medical University, Taipei, Taiwan; ^4^ Department of Dermatology, Taipei Medical University Shuang Ho Hospital, New Taipei City, Taiwan; ^5^ Department of Biochemistry and Molecular Cell Biology, School of Medicine, College of Medicine, Taipei Medical University, Taipei, Taiwan; ^6^ Graduate Institute of Medical Sciences, College of Medicine, Taipei Medical University, Taipei, Taiwan; ^7^ TMU Research Center of Cancer Translational Medicine, Taipei Medical University, Taipei, Taiwan; ^8^ Center for Reproductive Medicine, Taipei Medical University Hospital, Taipei Medical University, Taipei, Taiwan; ^9^ Comprehensive Cancer Center of Taipei Medical University, Taipei, Taiwan; ^10^ PhD Program for Translational Medicine, College of Medical Science and Technology, Taipei Medical University, Taipei, Taiwan

**Keywords:** vitiligo, autoimmune skin disorder, skin-resident memory T (TRM) cell, janus kinase, stem cell therapy

## Abstract

Vitiligo is a chronic autoimmune depigmenting skin disorder characterized by patches of the skin losing functional melanocytes. Multiple combinatorial factors are involved in disease development, among which immune T cells play a prominent role. The immune cells implicated in melanocyte destruction through adaptive immunity include CD8^+^ cytotoxic T cells and regulatory T cells, and aberrantly activated skin-resident memory T cells also play a role in melanocyte destruction. Over the past several years, major progress in understanding vitiligo pathogenesis has led to the development of targeted therapies. Janus kinase (JAK) inhibitors, which share the similar mechanism that autoactivates CD8^+^ T cells in chronic inflammatory diseases, have been reported to have therapeutic significance in vitiligo. Recently, immunomodulatory therapeutic interventions in vitiligo have been emerging. Mesenchymal stem cells (MSCs) regulate cytokine secretion and the balance of T-cell subsets, which makes them a promising cell-based treatment option for autoimmune diseases. The induction of MSC-mediated immunomodulation is complicated and occurs by contact-dependent mechanisms and soluble extracellular vesicle (EV) mediators. EVs released from MSCs contain various growth factors and cytokines with anti-inflammatory effects in the skin immune response. Here, we summarize and discuss the progress to date in targeted therapies that immunomodulate the niche environment of vitiligo, from the clinical trial of JAK inhibitors to the potential of MSCs and MSC-EVs. The available information was collected to highlight the need for further research into the treatment of vitiligo.

## Introduction

Vitiligo is an autoimmune skin disorder characterized by white patches of skin losing functional melanocytes, the pigment-producing cells of the skin. Vitiligo is a common skin disorder with an incidence rate of 0.1–2% worldwide, and it has no sex bias (Kruger and Schallreuter, 2012). Vitiligo has a significant impact on patients’ quality of life and self-esteem and predisposes them to an increased risk of sunburn and skin cancer.

**FIGURE 1:**
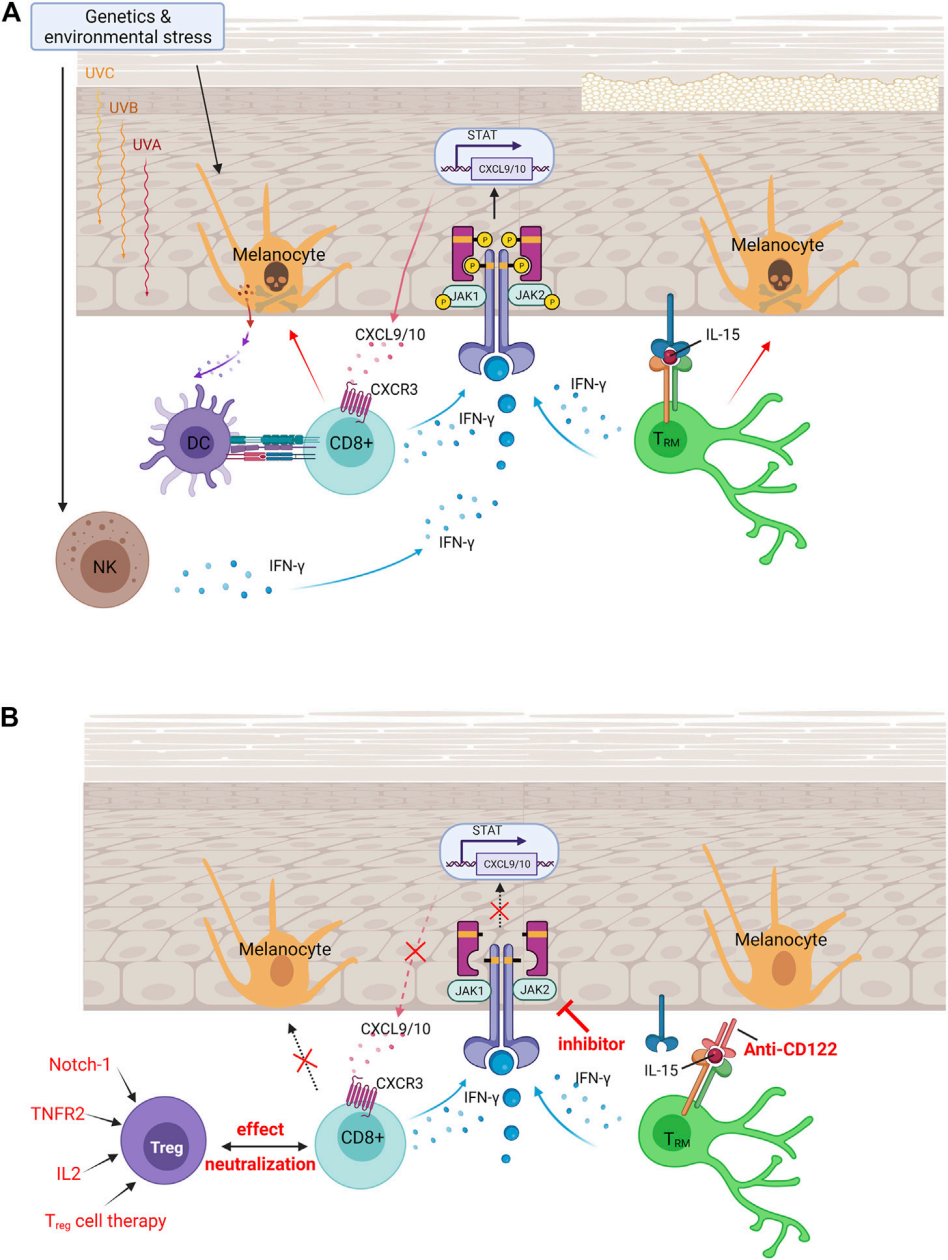
Vitiligo pathogenesis and currently available treatments for vitiligo. **(A)** Pathologies of vitiligo. In vitiligo, melanocytes are more susceptible to oxidative stress, which in turn targets antigens to nearby dendritic cells and induces their maturation into efficient antigen-presenting cells. Upon endogenous or exogenous stress, NK cells produce IFN-γ that induces the production of chemokines. Binding of IFN-γ to its receptor activates the JAK/STAT pathway and leads to T-cell recruitment and function, which induces melanocyte apoptosis. Established vitiligo is maintained by T_RM_ cells, which remain long-lived in the skin through IL-15–dependent signaling. **(B)** Immunomodulated therapeutic intervention in vitiligo. Immunomodulatory therapies are currently available treatments for vitiligo, including the increase of T_reg_ cells to neutralize effector CD8^+^ T-cell function, small molecule–targeted drugs of JAK inhibitors to block IFN-γ–CXCR3–CXCL9/10 signaling axis, and anti-CD122 antibody (IL-15 receptor subunit) to decrease IFN-γ production and deplete autoreactive CD8^+^ T_RM_ cells. DC, dendritic cells; NK, natural killer cells; T_RM_, skin-resident memory T cells; JAK, Janus kinase; IFN-γ, interferon-gamma; CXCL, chemokine (C-X-C motif) ligand; CXCR, chemokine (C-X-C motif) receptor; STAT, signal transducer and activator of transcription; T_reg_, regulatory T cells; IL-2, interleukin 2; IL-15, interleukin 15; TNFR2, tumor necrosis factor receptor 2.

Vitiligo is a multifactorial disorder that combines genetic susceptibility, the generation of inflammatory mediators from environmental triggers, and autoimmune responses (Figure 1) (Schallreuter et al., 2008; Bergqvist and Ezzedine, 2021). Attracted by proinflammatory cytokines, autoreactive CD8^+^ T cells that destroy the pigment-producing cells of melanocytes through the mediation of interferon-gamma (IFN-γ) signaling are believed to promote the apoptosis of melanocytes in vitiligo. Recent studies have found a subset of T cells called skin-resident memory T (T_RM_) cells, which do not circulate but reside at the white spot site of previous lesions. Therefore, T_RM_ cells are responsible for vitiligo relapse, the most difficult part of treatment.

The treatment of vitiligo is still one of the most difficult dermatological challenges. Actual treatments rely on the use of topical steroids or topical calcineurin inhibitors and are more effective when combined with phototherapy. However, relapse occurs in ∼40% of patients with vitiligo within 1 year after stopping treatment (Cavalie et al., 2015). Ideally, management of vitiligo focuses on stopping the immune destruction of melanocytes, halting depigmentation, stimulating repigmentation, and preventing relapses. Mesenchymal stem cells (MSCs) could induce melanocyte regeneration and have immunomodulatory properties that balance T-cell subsets (Weiss and Dahlke, 2019). Over the past several years, major progress in the understanding of vitiligo pathogenesis (Bergqvist and Ezzedine, 2020) and the application of MSC/other cellular therapies have led to the development of targeted therapies that are now being tested (Esquivel et al., 2020; Ha et al., 2020).

This review provides an update on the pathogenesis of vitiligo. We summarize the progress to date of MSC-based therapy and discuss its potential for new therapeutic interventions for the chronic autoimmune disease vitiligo.

## Pathogenesis of Immunity in Vitiligo

### Initiators of Oxidative Stress and Immunity

Oxidative stress is considered one of the most crucial initiators of vitiligo. Environmental factors such as ultraviolet radiation accelerate the generation of reactive oxygen species and activate the unfolded protein response (UPR), which initiate several inflammatory mediators. Under oxidative stress, UPR activation causes stressed melanocytes and stressed keratinocytes to release several inflammatory mediators that are reported to be biomarkers of vitiligo, such as interleukin (IL)-1β, C-X-C chemokine ligand 9 (CXCL9), CXCL10, and CXCL16. Stressed melanocytes release CXCL12 and CCL5, which are mediated in T-cell homing to the skin in vitiligo (Rezk et al., 2017). Stressed keratinocytes produce CXCL16, which recruits CD8^+^ T cells (Li et al., 2017). This proinflammatory factor further causes the activation of natural killer (NK) cells and chemoattracts melanocyte-specific CD8^+^ T cells to melanocytes and causes their apoptosis. Last, oxidative stress alters the WNT pathway, which is involved in melanocyte differentiation by decreasing WNT expression (Regazzetti et al., 2015).

By sensing stress signals released from melanocytes and keratinocytes, innate immune cells, including NK cells and dendritic cells (DCs), are activated early in vitiligo (Xie et al., 2016). Exosomes act as the mediator of communication between stressed melanocytes and immune cells (Wong et al., 2020). These exosomes deliver vitiligo target antigens to nearby DCs and stimulate the immune system. We discuss the details of the effect of exosomes later.

### Role of CD8^+^ T Cells

In vitiligo, adaptive immune activation is responsible for killing melanocytes specifically. Patchy infiltration of T cells occurs adjacent to melanocytes, especially in the leading edge of vitiligo depigmentation (van den Boorn et al., 2009). Serum frequency of melanocyte-specific CD8^+^ T cells is higher in patients with vitiligo than in their healthy counterparts, and the frequency is related to disease severity (Richmond et al., 2013).

CD8^+^ T cell mediates melanocyte apoptosis in various ways (Chen et al., 2021). CD8^+^ T cells produce several cytokines, including the proinflammatory cytokine IFN-γ. After the binding of IFN-γ to its receptor, the Janus kinase (JAK)/signal transducer and activator of transcription (STAT) pathway activates and stimulates the transcription of the chemokine ligands CXCL9 and CXCL10, which create a positive feedback loop for T-cell recruitment and function (Bergqvist and Ezzedine, 2021). Both CXCL9 and CXCL10 share a single receptor: CXCR3. Melanocyte-specific autoreactive T cells in patients with vitiligo express CXCR3 in both the blood and in lesional skin (Harris, 2016). In a mouse model, CXCR3-depleting antibodies reduce autoreactive T-cell numbers and reverses vitiligo (Richmond et al., 2017). Therefore, IFN-γ is thought to have one of the central roles to promote autoreactive CD8^+^ T-cell recruitment for vitiligo pathogenesis.

### Role of Skin-Resident Memory T Cells

Vitiligo is thought to be a skin memory disease because clinical observations show that relapse occurs essentially at the same location as a previously depigmented spot (Cavalie et al., 2015), implying that autoimmune memory plays a crucial role in the recurrence of vitiligo lesions.

Memory T cells were thought to be circulatory and to enter the tissues only to clear infection. Recent research has defined another pool of memory T cells called T_RM_ cells. T_RM_ cells do not circulate but reside in specific tissues to provide rapid protective immunity against reinfecting pathogens, and they recruit effector cells from the circulation (Jiang et al., 2012; Mueller and Mackay, 2016; Richmond et al., 2019). Of the memory T cells present in healthy skin tissue, 20–60% of the population are T_RM_ cells, revealing the high variation among healthy individuals (Boniface et al., 2018). In vitiliginous skin, the population of T_RM_ cells is high (up to 93%), consistent with their role as memory cells that persist after inflammation elimination. Functional CD8^+^ skin T_RM_ cells were found in vitiligo, suggesting that T_RM_ cells are responsible for long-term maintenance and potential relapse of vitiligo (Boniface et al., 2018; Richmond et al., 2019).

To facilitate the effect of T_RM_ cells, appropriate cytokine niches expressing IL-12, IL-15, IL-33, IFN-β, tumor necrosis factor (TNF)-α, and transforming growth factor (TGF)-β are formed in tissues (Mackay et al., 2013). Many studies have found that IL-15 supports T_RM_ cell development, maintenance, and survival (Mackay et al., 2015). IL-15–deficient mice showed reduced T_RM_ cell formation through the reduction of Bcl-2 expression, a prosurvival molecule, in CD103^+^ T_RM_ cells (Mackay et al., 2013). Both human and mouse autoreactive T_RM_ cells in vitiligo express high levels of the CD122 subunit of the IL-15 receptor in the blood and lesional skin. Melanocyte-specific T_RM_ cells cluster and produce high IL-15 levels in the vitiliginous skin (Malik et al., 2017), and antibody blockade of IL-15 signaling durably reverses depigmentation (Richmond et al., 2018). Therefore, IL-15 signaling was thought to be a therapeutic target for vitiligo.

### Role of Regulatory T Cells

Regulatory T cells (T_reg_ cells) are a subpopulation of T cells with tolerance to self-antigens, and they help prevent autoimmune diseases. T_reg_ cells isolated from the peripheral blood of patients with vitiligo are impaired, suppressing the proliferation and activation of CD8^+^ T cells *in vitro* (Lili et al., 2012). The exosomal pathway mediates the activation of CD8^+^ T cells, as well as the T_reg_ cells balance which is associated with the disruption of autoimmune tolerance in vitiligo (Wong et al., 2020). The expression of immune profile including transcription factor T-beta, TBX21 (T-Box Transcription Factor 21), CXCR3, and CCR5 (C-C Motif Chemokine Receptor 5) in T_reg_ is believed to support the formation of resident memory T cells (Ferreira et al., 2020). Current studies based on single-cell RNA sequencing of human vitiligo reveals that CCL5-CCR5 cytokine signaling serve as a chemokine circuit between effector CD8^+^T cells and T_reg_ cells (Gellatly et al., 2021). Therefore, modulating the immune response is considered as a therapeutic target for the treatment of vitiligo.

## Immunomodulatory Therapeutic Intervention in Vitiligo

Vitiligo is a chronic disease that requires lifelong therapy with immune mediators, phototherapy, or skin grafting. However, ∼40% of patients with vitiligo experience relapse within 1 year after stopping treatment (Cavalie et al., 2015). The advancement of technology has enabled the development of alternative target therapies for vitiligo. Recently, immunomodulation of JAK signaling, T_RM_ cells, and T_reg_ cells has become a promising treatment for vitiligo.

The effect of the IFN-γ–CXCL9/CXCL10–CXCR3 axis on the killing of melanocytes by CD8^+^ T cells is significant. In this axis, keratinocytes sense IFN-γ and generate CXCL9/CXCL10 mediated by JAK and associated STAT. JAK is a family of four proteins: JAK1, JAK2, JAK3, and TYK2. After stimulation by specific cytokines, JAKs form a heterodimer with various combinations, activating different STATs (Angelini et al., 2020). JAK inhibitors are emerging as a new class of small molecule–targeted drugs for the treatment of rheumatoid arthritis, a chronic inflammatory disorder that primarily affects joints. In dermatology, considerable progress has been made in emerging topical and systemic JAK inhibitor treatments (Solimani et al., 2019). A report showed that a patient who had both alopecia areata, a common immunological cause of hair loss, and vitiligo experienced some hair regrowth and repigmentation after treatment with a JAK inhibitor targeting JAK1/2, ruxolitinib (Harris et al., 2016). This is a potential therapy for vitiligo because such chronic inflammatory disorders exhibit similar pathogenesis, as they are both IFN-γ–driven and dependent on CD8^+^ T cells (Bertolini et al., 2020). The latest phase 2 trial showed that the JAK inhibitor ruxolitinib was effective in vitiligo treatment, with high clinical relevance and therapeutic significance (Rosmarin et al., 2020). Another pan-JAK inhibitor, tofacitinib, also shows repigmentation ability when combined with phototherapy for stimulating melanocytes, providing a higher treatment effect (Liu et al., 2017). The literature has shown that the most significant repigmentation is seen on the face, whereas patches located on the trunk or lower extremities are not prominent. This raises an interesting issue regarding the regional specificity of the vitiligo lesion site, some of which is in a bilateral symmetric distribution (Bergqvist and Ezzedine, 2021). Another point worth noting is that much of the regained pigment regressed after discontinuing ruxolitinib, whereas much of the regrown hair was maintained (Harris et al., 2016). This indicates that although these autoimmune diseases exhibit similar pathogenesis, JAK inhibitors exert different effects on clinical manifestations. On the basis of the complexity of JAK signaling, toxicities that may limit a strong and ubiquitous JAK blockade should be considered.

The rapid, but not durable, repigmentation effect of JAK inhibitors indicates the most troublesome recurrence symptoms of vitiligo. JAK inhibitors exert their action by abrogating the chemotaxis of cytotoxic cells instead of by removing T_RM_ cells that remain long-lived in the skin through IL-15–dependent signaling. Subsequent studies have therefore investigated therapeutic treatment targeting IL-15 signaling. Treatment with anti-CD122 antibody, a subunit of the IL-15 receptor on human and mouse T_RM_ cells, has been shown to decrease IFN-γ production and to deplete autoreactive CD8^+^ T_RM_ cells in mice with established vitiligo (Richmond et al., 2018).

T_reg_ cells have immunosuppressive properties; hence, their expansion may be an efficient strategy. T_reg_ expansion can be achieved through ectopic expression of its regulators IL-2, TNF receptor 2, and Notch-1 (Mukhatayev et al., 2021). IL-2 is required for the differentiation of T_reg_; conversely, T_reg_ expresses the high-affinity IL-2 receptor to collect IL-2. TNF receptor 2 is highly expressed on T_reg_ cells, and, in graft-versus-host disease, stimulated TNF receptor 2 is reported to efficiently expand natural T_reg_ cells. Notch-1 inhibition enhances T_reg_ populations and suppressive function in transplantation. Another possible option is T_reg_ cell therapy, in which T_reg_ cells are harvested from the patient’s circulation to expand them *in vitro* and transfer the expanded T_reg_ cells back to the patient. The challenge is the scarcity of T_reg_ cells in the blood and the slow rate of expansion *in vitro* when applied in clinical treatment (Sakaguchi et al., 2020). To improve the number of T_reg_ cells, elevation of the recruitment of T_reg_ cells to the epidermis through gene gun treatment–induced CCL22 overexpression is the currently preferred option to delay vitiligo progression (Eby et al., 2015). To improve the quality of T_reg_ cells, the emerging method available is chimeric antigen receptor (CAR) T cell therapy. Modified T_reg_ cells are engineered *in vitro* to recognize a specific target antigen and are then returned to the patient to stimulate intracellular signaling to increase the T-cell response to gain higher function than the nonspecific bystander T_reg_ cells (Mukhatayev et al., 2021). With the help of CAR technology, abundant antigen-specific T_reg_ cells can be acquired through *in vitro* expansion, and after adding specificity to T_reg_ cells, T cells with specificity can be transformed into T_reg_ cells (Rosado-Sanchez and Levings, 2020). However, the transduced T_reg_ cells may still function as cytotoxic T cells due to instability. Further technological developments may improve the quality and reliability of the CAR T_reg_ cell production for clinical application.

## Immunomodulatory Effects of MSCs and Their Extracellular Vesicles

### Mesenchymal Stem Cell

**FIGURE 2:**
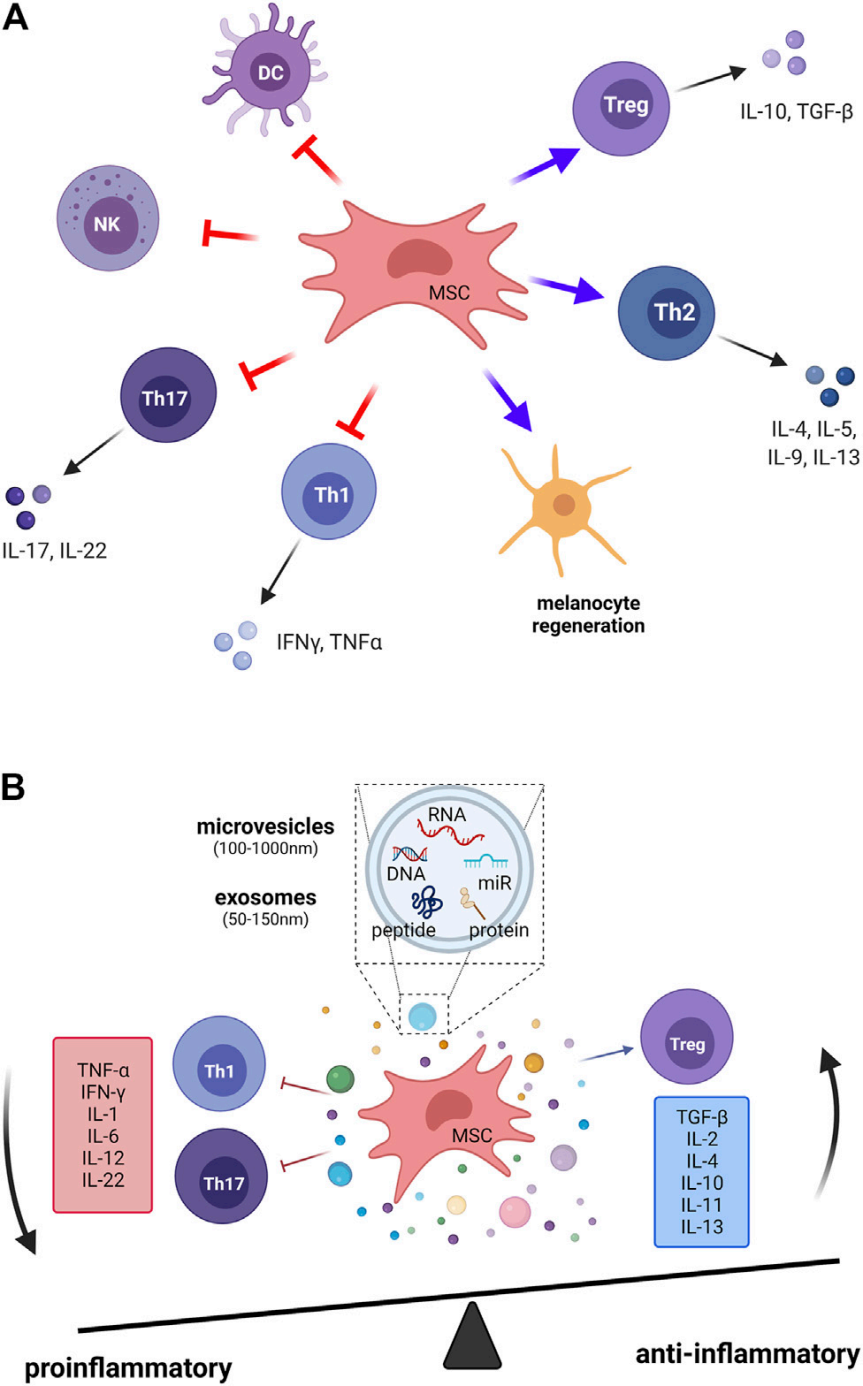
The immunomodulatory effects of mesenchymal stem cells in vitiligo. The induction of MSC-mediated immunomodulation is complicated through **(A)** cell–cell contact–dependent mechanisms and **(B)** several soluble mediators that are secreted from MSCs. Treg, regulatory T cells; Th1, T helper 1 cell; Th2, T helper 2 cell; Th17, T helper 17 cell; DCs, dendritic cells; NK, natural killer cells; MSC, mesenchymal stem cell; IL, interleukin; IFN-γ, interferon-gamma; TNF-α, tumor necrosis factor-alpha; TGF-β, transforming growth factor-beta.

MSCs show promise as a type of cell-based therapy option for autoimmune diseases based on their immunomodulatory properties. We summarize the clinical trials of cell therapy in autoimmune-related skin diseases (Supplementary Table S1). The induction of MSC-mediated immunosuppression is complicated and occurs through contact-dependent mechanisms and several soluble mediators (Figure 2). Although the exact mechanisms that mediate the immunomodulatory effects of MSCs are still not fully understood, cell-based MSC therapy remains prevalent because MSCs can be applied in patients with vitiligo in several ways, including stopping immune destruction, stimulating repigmentation, and preventing relapses.

Studies have demonstrated that MSCs suppress immune reactions and mediate complicated mechanisms that change the T-cell subset polarization from proinflammatory (Th1, Th17) subsets to anti-inflammatory Th2 (Lu et al., 2009; Duffy et al., 2011) and T_reg_ cells (Ghannam et al., 2010). On the one hand, in MSC-treated animals, the levels of inflammatory cytokines, including IL-17, IFN-γ, IL-2, and TNF-α, produced by Th1 and Th17 are significantly reduced. On the other hand, the anti-inflammatory cytokines IL-10 and TGF-β are highly expressed on T_reg_ cells, which directly suppress responder T cells (Chatterjee et al., 2014; Ujiie, 2019). These cytokines lead to tissue damage and vitiligo pathology. To sum up, MSCs regulate cytokine secretion and the balance of T-cell subsets, which stop the immune destruction of melanocytes.

Regarding the cell–cell interaction aspect, DC and NK cells are also important targets for the immunomodulatory activity of MSCs. MSCs affect the differentiation, migration, maturation, and antigen presentation of DCs, which leads to triggering of tolerogenic T cells (Jiang et al., 2005; English et al., 2008). Adult MSCs inhibit IL-2–induced NK cell activation (Spaggiari et al., 2006).

In addition to their immunomodulatory properties, MSCs promote human melanocyte proliferation and resistance to apoptosis through the PTEN pathway in vitiligo (Zhu et al., 2020). It has been reported that dermal mesenchymal stem cells (DMSCs) could significantly inhibit the skin-homing activity of CD8^+^ T lymphocytes (Zhou et al., 2013). Clinical trial shows that transplantation of cell suspension containing DMSCs resulted in excellent response in patients with vitiligo to extent of repigmentation (Thakur et al., 2019). Adipose tissue-derived mesenchymal stem cells (ADSCs) have a multipotential nature that can differentiable into melanocytes under the presence of beta FGF and melanocyte growth factor (Zhang P. et al., 2014). The potential application of ADSCs in vitiligo can take the advantages from that the ADSCs can increase the proliferation of melanocytes under the presence of beta FGF and melanocyte growth factor, inhibit the production of proinflammatory cytokines, and protect against apoptosis (Owczarczyk-Saczonek et al., 2017).

MSCs isolated from different regions show phenotypic heterogeneity (Chen et al., 2020). To achieve their therapeutic benefit, a careful evaluation of appropriate cell sources and consistent protocols and dosages should be conducted in the future.

### Extracellular Vesicles

Current evidence shows that in soluble mediators, the released extracellular vesicles (EVs) from MSCs exert immunomodulatory effects. EVs are heterogeneous lipid bilayer–surrounded vesicles, and they act as mediators of intercellular communication. The recognition of exosomes by target cells is specific and occurs through surface receptors. MSCs derived EVs have shown the immunosuppressive role on different immune cells, including B cells, T cells, dendritic cells, and macrophages (Gomzikova et al., 2019). MSC exosomes could induce anti-inflammatory cytokines expression, and could activate M2-like monocytes to induced T_reg_ polarization (Zhang B. et al., 2014). Morrison et al. found that MSC derived EVs promote an anti-inflammatory and highly phagocytic macrophage phenotype via extracellular vesicle mitochondrial transfer (Morrison et al., 2017). Recent studies have reported that EVs are associated with skin physiology in the cutaneous microenvironment. Adipocyte-derived stem cell–conditioned media can activate hair growth, and microinjury using a fractional laser or microneedling can also induce wound healing and hair regeneration (Lee et al., 2021). The significantly reduction of mRNA expression of various pro-inflammatory cytokines in alleviate atopic dermatitis skin lesions on mice model by the ADSC-derived exosomes (Cho et al., 2018) provides the potential cell-free therapy of ADSC-derived exosomes in other immunological skin disease, such as vitiligo. We summarize the studies of extracellular vesicles for autoimmune-related skin diseases (Supplementary Table S2). Here, we highlight two aspects of the role of exosomes in vitiligo: the immunomodulation effects and the interaction of melanocytes and keratinocytes through exosomes (50–150 nm of EVs). First, the exosomal pathway is crucial for the regulation of CD8^+^ T cells and T_reg_ cells (Wong et al., 2020), which is key to the pathological conditions for vitiligo. Studies of human umbilical cord MSC-derived exosomes have shown the inhibition of the proliferation of CD8^+^ T cells and the increase in the proportion of T_reg_ cells (Liu et al., 2015). Exosomes derived from multiple sclerosis can disrupt T_reg_ cell homeostasis by suppressing the insulin-like growth factor 1 receptor and TGF-β receptor 1 (Kimura et al., 2018). The conditioned media from human umbilical cord blood–derived MSCs contain various growth factors and cytokines with anti-inflammatory effects in the skin immune response, as analyzed by RT-PCR and ELISA (Kim et al., 2020). Second, EVs function as a new mode of signaling in intercellular communication through carrying mediator vesicles (Stahl and Raposo, 2019). Both melanocytes and keratinocytes can secrete exosomes for mutual interaction. Synthesis of melanin in melanocytes occurs in organelles called melanosomes, and melanin is distributed to the epidermis through the transport of melanosomes to adjacent keratinocytes for skin pigmentation through exosomes (D'Mello et al., 2016). Furthermore, exosomes carrying selected microRNAs from murine keratinocytes are targeted to melanocytes and induce the inhibition of melanogenesis by altering gene expression and enzyme activity in melanocytes (Kim et al., 2014; Liu et al., 2019). As intercellular communicators, signaling EVs must interact with the target cell with high fidelity. However, little is known about how signals are transmitted to the intracellular target. More investigation on the specific target fidelity of EVs will improve the therapeutic strategies for vitiligo and other skin regenerative medicine interventions.

## Discussion

Vitiligo is a chronic depigmenting skin disorder, for which current management strategies have limited efficacy, emphasizing the need for improved treatment options. Similar to other autoimmune diseases, including rheumatoid arthritis in which immune cells attack joints, and alopecia areata that attacks the hair bulb, immune cells target melanocytes in vitiligo through similar immune cell populations and cytokines to activate the JAK signaling pathway. JAK inhibitors are emerging as a new class of small molecule–targeted drugs for the treatment of chronic inflammatory diseases, including rheumatoid arthritis, alopecia areata, and vitiligo. The latest phase 2 trial showed that ruxolitinib cream was effective in vitiligo treatment, with high clinical relevance and therapeutic significance (Rosmarin et al., 2020). Although the value of different JAK inhibitor specificities across disease states remains to be defined, JAK inhibitors are an effective and safe alternative treatment for vitiligo.

Compared with previous relatively rudimentary treatment with immuosuppressants or steroids that have various side effects and with which relapse is common, novel immunomodulatory therapies can balance the immune mechanisms by changing stem cells. Stem cells have anti-inflammatory and immunomodulatory properties and have potential as a targeted therapy for vitiligo. MSC-derived extracellular vesicles, which accompany exosomes that act as potent drug carriers by delivering cargo to target cells, are an emerging management approach. Moreover, because MSCs often require invasive procedures, approaches that only require them to be cultured *in vitro* and use of their released EVs may have increased scalability and yield per MSC batch. EVs are easier to preserve and transfer, have lower immunogenicity, and therefore are safer for therapeutic administration.

We propose that the method of MSCs administration depends on the lesional pattern of Vitiligo. Generalized pattern is the common type of vitiligo (80–95%) in which multiple spots on the skin that are found on both sides of the body. Segmental vitiligo affects only one area of the body, which affects about 5% of adults and 20% of children. In the case of generalized vitiligo, intravenous administration could be applied because MSCs can affect systematically. It has been reported that MSCs migrate in response to inflammatory mediators and home to injured sites (Chapel et al., 2003; Zhuang et al., 2021). The supported examples could be found when using ADSCs to treat chronic wound (phase I, NCT02961699). In the case of segmental vitiligo, administrated MSCs can be given on the lesion site by intradermal injection (Kandil, 1970), microneedling, or spray-on (Supplementary Table S1). Depending on different considerations, there may have several ways for MSC-derived extracellular vesicles administration, including intravenous injection and inhalation for systematic effects, as well as *in situ* injection or ointment for local lesion sites. The biodistribution of administrated MSCs is important for the safety and efficacy of cell therapy. Several factors that can affect the pharmacokinetics of the administered MSCs, including cell size, cell source, immunological features, and immunogenic reactions (Zhuang et al., 2021). Future directions will be focused on the real-world efficacy and safety of this new class of immunomodulatory therapies.
