# Supplementary material for: Vitiligo: An Autoimmune Skin Disease and its Immunomodulatory Therapeutic Intervention
Source: Front Cell Dev Biol. 2021 Dec 14;9:797026. doi: 10.3389/fcell.2021.797026 (PMC8712646; doi:10.3389/fcell.2021.797026)
Supplement: Supplementary file 1 [file DataSheet2.PDF]

Table 2. Application of extracellular vesicles for autoimmune-related skin diseases

Source of EVs	Model of disease	Effective Molecule	Therapeutic Use	References
ADSCs	Atopic Dermatitis	reduction of expression of inflammatory cytokines (IL-4, IL-23, IL-31 and TNF-α)	reduction of symptoms	(Boniface and Seneschal, 2019)
Macrophages	Alopecia	Wnt3a and Wnt7b proteins	promotes hair growth	(Rajendran et al., 2020)
Urine	Systemic lupus erythematosus (SLE)	miR-146a	biomarker	(Perez-Hernandez et al., 2015)
Blood	Systemic lupus erythematosus (SLE)	IgG, IgM, and C1q	biomarker	(Nielsen et al., 2012)
Blood	Systemic lupus erythematosus (SLE)	miR-21, miR-155	biomarker	(Li et al., 2020)
Blood	Psoriasis	IL-17	biomarker	(Jacquin-Porretaz et al., 2019)
Blood	Psoriasis	miR-199a	biomarker	(Wang et al., 2021)
Blood	Psoriasis	miR-151a, miR-199a, miR-370, miR-589, and miR-769	biomarker	(Chen et al., 2019)
Saliva	Lichen planus	miR-1246, miR-1290, and miR-4484	biomarker	(Byun et al., 2015)
