# Supplementary material for: Vitiligo: An Autoimmune Skin Disease and its Immunomodulatory Therapeutic Intervention
Source: Front Cell Dev Biol. 2021 Dec 14;9:797026. doi: 10.3389/fcell.2021.797026 (PMC8712646; doi:10.3389/fcell.2021.797026)
Supplement: Supplementary file 2 [file DataSheet1.PDF]

Table 1. The clinical trials of cell therapyies in autoimmune-related skin diseases.

NCT no.	Types of cell therapy *	Resourse	Route	Phase	Disease	Status	Autograft/ Allograft	Year
Vitiligo								
NCT03013049	Dermal-MSCs	Skin	Spreading	NA	Vitiligo	Unknown	Autograft	2017
NCT00631865	Melanocytes	Skin	Epidermis injection	3	Vitiligo	Completed	Autograft	2005
NCT01923142	Melanocytes	Plucked hair follicles	--	2	Vitiligo	Terminated	Autograft	2013
NCT03022019	Epidermal cells	Skin	--	NA	Vitiligo	Terminated (Futility)	Autograft	2017
NCT03497208	Melanocytes and keratinocytes	Skin	Microneedling	NA	Vitiligo	Unknown	Autograft	2018
NCT04271501	Melanocytes and Keratinocytes	Skin	--	NA	Vitiligo	Recruiting	Autograft	2020
NCT04547998	Skin cells	Skin	Spray-on	NA	Vitiligo	Recruiting	Autograft	2020
Alopecia Areata								
NCT03078686	AD-cSVF + AD-tSVF + PRP	Adipose	Intravenous infusion	NA	Alopecia Areata and	Recruiting	--	2017
NCT03427905	AD-MSCs	Adipose	Scalp injection	NA	Alopecia	Unknown	Autograft	2018
NCT01673789	Educated lymphocytes	Blood	Infusions	1/2	Alopecia Areata	Unknown	Autograft	2012
NCT04011748	Educated mononuclear cells	Blood	Infusions	2	Alopecia Areata	Not yet recruiting	Autograft	2019
Atopic Dermatitis								
NCT01927705	UCB-MSCs	Umbilical cord Blood	--	1/2	Atopic Dermatitis	Completed	Allograft	2013
NCT04725136	UCB-MSCs	Umbilical cord Blood	Subcutaneous injection	1/2	Atopic Dermatitis	Enrolling by invitation	Allograft	2021
NCT03269773	UCB-MSCs	Umbilical cord Blood	Injected respectively into both upper arms, both thighs, and abdomen (5 regions)	3	Atopic Dermatitis	Completed	Allograft	2017
NCT04137562	AD-MSCs	Adipose	Injected intravenously	2	Atopic Dermatitis	Recruiting	--	2019
Psoriasis								
NCT03265613	AD-MSCs	Adipose	Infusions	1/2	Psoriasis	Active, not recruiting	Allograft	2017
NCT03392311	AD-MSCs	Adipose	Infusions	1/2	Psoriasis	Enrolling by invitation	Allograft	2018
NCT04785027	AD-MSCs	Adipose	Infusions	1/2	Psoriasis	Recruiting	Allograft	2021
NCT03765957	UC-MSCs	Umbilical cord	Intravenous infusion	1	Psoriasis	Recruiting	Allograft	2018
NCT03424629	UC-MSCs	Umbilical cord	Infusions	1	Plaque Psoriasis	Unknown	Allograft	2018
NCT02491658	UC-MSCs	Umbilical cord	Infusions	1/2	Psoriasis Vulgaris	Unknown	Allograft	2015
Others								
NCT02213705	MSCs	--	Injection	1/2	Systemic Scleroderma	Active, not recruiting	Allograft	2014
NCT04356287	UC-MSCs	Umbilical cord	Infusions	1/2	Systemic Sclerosis	Not yet recruiting	Allograft	2020
NCT05029336	CD3/CD19-depleted hematopoietic stem cell	--	--	2	Systemic Lupus Erythematosus and Systemic Sclerosis	Recruiting	Autograft	2021
NCT04684602	Amniotic and umbilical cord stem cell rich tissue substance	Amniotic and umbilical cord	--	1/2	Autoimmune Diseases, Integumentary	Recruiting	Allograft	2020
NCT02824393	AD-MSCs	Adipose	Intravenous infusion	1	Urticaria	Completed	Autograft	2016

* MSCs: Mesenchymal stem cells; AD-cSVF: Adipose-derived cellular stromal vascular fraction; AD-tSVF: Adipose-derived tissue stromal vascular fraction; PRP: Platelet rich plasma.
